# High concentrations of mast cell chymase facilitate the transduction of the transforming growth factor-β1/Smads signaling pathway in skin fibroblasts

**DOI:** 10.3892/etm.2015.2216

**Published:** 2015-01-26

**Authors:** XIANGLIN DONG, CHUANSHAN ZHANG, SHAOLIN MA, HAO WEN

**Affiliations:** 1 Department of Burns and Plastic Surgery, Clinical Medical Research Institute, The First Affiliated Hospital of Xinjiang Medical University, Ürümqi, Xinjiang 830011, P.R. China; 2 State Key Laboratory Incubation Base of Xinjiang Major Diseases Research, Clinical Medical Research Institute, The First Affiliated Hospital of Xinjiang Medical University, Ürümqi, Xinjiang 830011, P.R. China

**Keywords:** mast cell chymase, fibroblast, transforming growth factor-β1, Smads

## Abstract

The aim of the present study was to investigate the effect of different concentrations of mast cell chymase on the transforming growth factor (TGF)-β1/Smad signaling pathway in skin fibroblasts. Cultured skin fibroblasts were treated with various concentrations of chymase for different time periods. A 3-(4,5-dimethylthiazol-2-yl)-2,5-diphenyltetrazolium bromide assay was used to assess the rate of cell proliferation. In addition, protein expression in the fibroblasts was measured using western blot analysis. Chymase was shown to enhance the proliferation of skin fibroblasts following incubation for 48, 72 and 96 h (P<0.01). Furthermore, high concentrations of mast cell chymase were shown to enhance the mRNA and protein expression levels of TGF-β1 after long-term (≥6 h) incubation. In addition, high concentrations of mast cell chymase increased P-Smad2/3 and Smad2/3 protein expression. By contrast, low concentrations of mast cell chymase increased Smad7 protein expression. Therefore, the results demonstrated that high concentrations of mast cell chymase facilitated the transduction of the TGF-β1/Smad signaling pathway in skin fibroblasts.

## Introduction

Skin wound healing is one of the most complex biological processes and is affected by multiple factors. The process involves three steps of inflammation, cell proliferation and tissue remodeling, in order to maintain the body homeostasis (1). This complex process is regulated by a signaling network system with the same complexity, which includes numerous growth factors, cytokines and chemokines (2,3). Following activation, transforming growth factor (TGF)-β, a key regulator in wound healing, can stimulate extracellular matrix deposition and angiogenesis by regulating the proliferation of fibroblasts (2,4). The action of TGF-β is accomplished through the Smad signaling pathways (5).

Autophosphorylation of TGF-β occurs after binding to the heterodimer receptor complex, which activates the downstream transcription factor signaling molecules that belong to the Smad protein family (2,3). Following activation, TGF-β receptors induce the phosphorylation of Smad2 and Smad3, forming hetero-oligomeric complexes with Smad4 (6–8). The complexes are transported into the nucleus where they regulate ligand-induced gene transcription. The signaling pathway following the activation of the TGF-β receptor is further regulated by Smad7, which functions as an intracellular antagonist (9). Smad7 can firmly bind to the activated TGF-β1 receptor, inhibit the phosphorylation of R-Smad and hence inhibit the signaling pathway (9).

Mast cell chymase is a type of serine proteinase that is included in the secretory granules of mast cells. Following activation, mast cell chymase can transform inactivated TGF-β1 into an activated form (10,11). Taipale *et al* (12) demonstrated that in the extracellular matrix of human epithelial and endothelial cells, chymase facilitates the release of TGF-β1 from the bound protein. Mast cell chymase can increase the concentration of TGF-β1 in cultured fibroblasts; however, the increase in TGF-β1 can be attenuated using a chymase inhibitor (13). In addition, a neutralizing antibody of TGF-β1 has been shown to completely inhibit chymase-induced fibroblast proliferation, indicating that chymase promotes cell proliferation through TGF-β1 (13). Furthermore, chymase regulates the formation of angiotensin II (Ang II) (14), degrades procollagen in tissue remodeling (15,16) and participates in inflammatory responses (17,18).

Mast cells originate from bone marrow CD34^+^ hematopoietic stem cells, and are distributed to various tissues via the blood circulation. Mature mast cells are only found in tissues, and those found in the blood are precursors. Previous studies have revealed that chymase promotes the proliferation of skin fibroblasts in a dose- and time-dependent manner (19), and chymase may be involved in the wound healing process (20,21). Chymase is known to activate TGF-β1 (10,11), which plays a central role in wound healing and fibrosis (22). In addition, chymase has been reported to induce myocardial fibrosis via the activation of the TGF-β1/Smad signaling pathway (23). However, the effect of chymase on the TGF-β1/Smad signaling pathway in skin fibroblasts remains unknown. In the present study, the effects of different concentrations of mast cell chymase were investigated on the TGF-β1/Smad signaling pathway in skin fibroblasts.

## Materials and methods

### Cell culture

Skin tissues were obtained from patients treated at the Department of Burns and Plastic Surgery in the First Affiliated Hospital of Xinjiang Medical University (Ürümqi, China). The collection and use of tissue samples were approved by the Ethics Committee of the First Affiliated Hospital of Xinjiang Medical University. Written informed consent was obtained from all the participants.

Skin tissues were cut in sterile conditions and placed into phosphate-buffered saline containing 100,000 U/l penicillin and 100 mg/l streptomycin. After soaking for 30 min, the tissues were transferred to Petri dishes where the subcutaneous tissues were eliminated, and the samples were cut into small strips. The tissues were digested with 0.25% Dispase II (Sigma-Aldrich, St. Louis, MO, USA) at 4°C overnight. After removing the epidermis, the isolated dermis was cut into sections of 1–2 mm^3^. The tissue sections were digested at 37°C with shaking for 3 h. The filtrate was collected via a 150 μm mesh (Tiantai Global Screen Mesh Co., Ltd., Taizhou, China), and the remainder was centrifuged at 1,000 × g for 10 min to collect the cells. The cells were seeded onto Petri dishes at a density of 2×10^4^ cells/cm^2^ and cultured at 37°C in the presence of 5% CO_2_. The medium was changed after 4 h incubation, following which the medium was changed every three days. Cell growth and shapes were observed under an inverted microscope (BX50; Olympus, Tokyo, Japan). The third to sixth generations of the cells were used for further study.

### 3-(4,5-dimethylthiazol-2-yl)-2,5-diphenyltetrazolium bromide (MTT) assay

Cell proliferation was analyzed using an MTT assay. Cultured fibroblasts were trypsinized, made into a single cell suspension (1×10^6^ cells/ml) and seeded onto 96-well plates for incubation for 24 h. The cells were divided into five groups for the addition of different concentrations (0, 15, 30, 60 and 120 ng/ml) of chymase (C8118; Sigma-Aldrich). The five groups of cells were cultured for 24, 48, 72 and 96 h, followed by the addition of 20 μl MTT (0.5%) per well prior to continued culture for an additional 4 h. The supernatants were discarded, and 100 μl dimethyl sulfoxide was added to each well prior to shaking for 10 min. Optical density (490 nm) values were measured using a microplate reader (Thermo Plate TP-Reader; Thermo Fisher Scientific, Waltham, MA, USA). All the experiments were performed in triplicate.

### Quantitative polymerase chain reaction (qPCR)

Skin fibroblasts were cultured in the presence of different concentrations (0, 15, 30, 60 and 120 ng/ml) of chymase for 6, 12 and 24 h. Following cell culture, the total RNA was isolated using TRIzol reagent (Invitrogen Life Technologies, Carlsbad, CA, USA). qPCR was conducted using SYBR Premix Ex Taq™ (Takara Bio, Inc., Otsu, Japan) on an IQ5 qRT-PCR system (Bio-Rad Laboratories, Hercules, CA, USA). PCR amplification conditions were as follows: Initial denaturation at 95°C for 30 sec, followed by 40 cycles of amplification at 95°C for 5 sec, 55°C for 30 sec and 72°C for 60 sec. The temperature range for the dissolution curve was 65–95°C. The 2^−ΔΔCt^ method was used to calculate the gene expression levels of TGF-β1 relative to glyceraldehyde-3-phosphate dehydrogenase (GAPDH). The sequences of the specific primers were as follows: TGF-β1 (158 bp), 5′-ACACCAACTATTGCTTCAG-3′ (sense) and 5′-TGTCCAGGCTCCAAATG-3′ (antisense); GAPDH (137 bp), 5′-GCACCGTCAAGGCTGAGAAC-3′ (sense) and 5′-TGGTGAAGACGCCAGTGGA-3′ (antisense). Each PCR trial was performed with three samples and repeated a minimum of three times.

### Bicinchoninic acid (BCA) assay

Cells were seeded into six-well plates at a density of 5×10^4^ cells/well. The cells were incubated with various concentrations of chymase for 6, 12 and 24 h. Following incubation, the cells were washed with ice-cold phosphate-buffered saline, and lysed in 500 μl ice-cold radioimmunoprecipitation assay buffer (BioTeke Corporation, Beijing, China), containing 1 μg/ml phosphatase inhibitors and 1 mM phenylmethanesulfonyl fluoride, for 30 min. The mixture was centrifuged at 12,000 × g (4°C) for 10 min, after which the supernatants were stored at −80°C. The concentration of TGF-β1, phosphorylated Smad2/3 (P-Smad2/3), Smad2/3, Smad7 and GAPDH proteins were measured using a BCA protein assay kit (BioTeke Corporation).

### Western blot analysis

A prestained marker with a low molecular weight and 30 μg protein from the tissue samples were subject to sodium dodecyl sulfate polyacrylamide gel electrophoresis. The separated proteins were transferred onto polyvinylidene fluoride membranes (EMD Millipore, Billerica, MA, USA) and blocked in Tris-buffered saline and Tween-20 [10 mM Tris (pH 7.6), 150 mM NaCl and 0.1% Tween-20] containing 5% skimmed milk for 1 h at room temperature. Subsequently, the membranes were incubated with primary antibodies against TGF-β1 (sc-146; 1:300; Santa Cruz Biotechnology, Inc., Santa Cruz, CA, USA), P-Smad2/3 (8828s; 1:1,000; Cell Signaling Technology, Inc., Beverly, MA, USA), Smad2/3 (sc-8332; 1:300; Santa Cruz Biotechnology, Inc.), Smad7 (sc-11392; 1:300; Santa Cruz Biotechnology, Inc.) and GAPDH (sc-25778; 1:300; Santa Cruz Biotechnology, Inc.) at 4°C overnight. The blots were rinsed in Tris-buffered saline and Tween-20, and incubated with an alkaline phosphatase-conjugated secondary antibody (77054s; 1:1,000; Cell Signaling Technology, Inc.) for 2 h at room temperature. Bands on the western blots were visualized using a 5-bromo-4-chloro-3-indolyl phosphate/nitroblue tetrazolium kit (Invitrogen Life Technologies), according to the manufacturer’s instructions. Optical densities of the bands were scanned and quantified using Quantity One image analysis software (Bio-Rad Laboratories).

### Statistical analysis

All statistical analyses were performed using SPSS 17.0 software for Windows (SPSS, Inc., Chicago, IL, USA). Data are presented as the mean ± standard deviation. Statistical differences between groups were assessed by one-way analysis of variance, followed by the least significant difference post hoc test. P<0.05 was considered to indicate a statistically significant difference.

## Results

### Mast cell chymase increases skin fibroblast proliferation in a dose- and time-dependent manner

**Figure 1:**
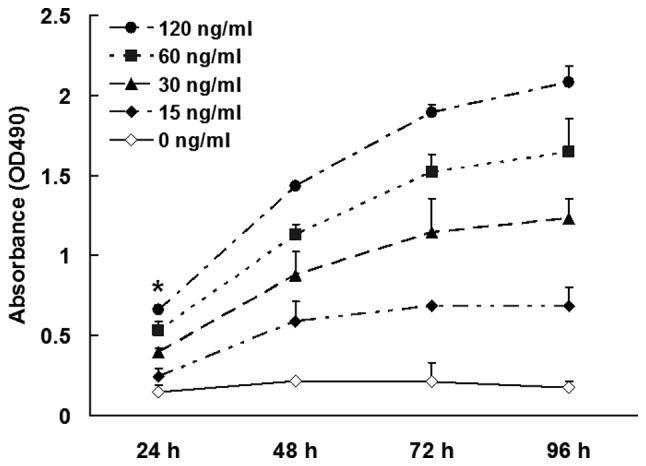
Proliferation of the skin fibroblasts was analyzed using an 3-(4,5-dimethylthiazol-2-yl)-2,5-diphenyltetrazolium bromide (MTT) assay. Cultured fibroblasts were trypsinized, made into a single cell suspension (1×10^6^ cells/ml) and seeded onto 96-well plates for incubation for 24 h. The cells were divided into five groups for the addition of different concentrations (0, 15, 30, 60 and 120 ng/ml) of chymase and cultured for 24, 48, 72 and 96 h, followed by the addition of 20 μl MTT (0.5%) per well and continued culture for an additional 4 h. OD (490 nm) values were measured and the experiments were performed in triplicate. Data are expressed as the mean ± standard deviation. ^*^P<0.01, vs. other groups. OD, optical density.

To analyze the effect of different concentrations of mast cell chymase on the proliferation of skin fibroblasts following incubation for 24, 48, 72 and 96 h, an MTT assay was performed. The results revealed that chymase (15, 30, 60 and 120 ng/ml) enhanced the proliferation of skin fibroblasts following incubation for 48, 72 and 96 h (P<0.01). As the incubation time increased, the proliferation was enhanced (Fig. 1). These results indicated that mast cell chymase increased skin fibroblast proliferation in a dose- and time-dependent manner.

### High concentrations of mast cell chymase enhance the mRNA and protein expression levels of TGF-β1 after long-term incubation

**Figure 2:**
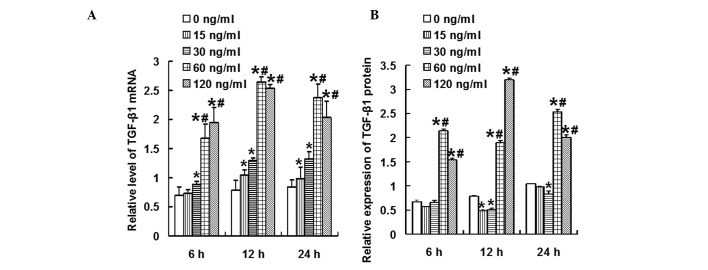
(A) mRNA and (B) protein expression levels of TGF-β1 in skin fibroblasts treated with chymase (0, 15, 30, 60 and 120 ng/ml) for 6, 12 and 24 h, as determined using quantitative polymerase chain reaction and western blot analysis, respectively. Data are expressed as the mean ± standard deviation. ^*^P<0.05, vs. the 0 ng/ml concentration group; ^#^P<0.05, vs. the 15 and 30 ng/ml groups. TGF, transforming growth factor.

To analyze the effect of various concentrations of mast cell chymase on the mRNA and protein expression levels of TGF-β1 after incubation for 6, 12 and 24 h, qPCR and western blot analysis were employed, respectively. The qPCR results revealed that the mRNA expression levels of TGF-β1 following incubation with chymase at concentrations of 60 and 120 ng/ml for 6, 12 and 24 h were significantly higher compared with those incubated with chymase at concentrations of 0, 15 and 30 ng/ml (P<0.01; Fig. 2A). In addition, western blot analysis demonstrated that TGF-β1 protein expression following incubation with chymase at 60 and 120 ng/ml for 6, 12 and 24 h was significantly higher compared with that in the control group (0 ng/ml; P<0.01). However, TGF-β1 protein expression levels following incubation with 15 and 30 ng/ml chymase were slightly lower compared with that in the control group (0 ng/ml; Fig. 2B). These results indicated that high concentrations of mast cell chymase (60 and 120 ng/ml) enhanced the mRNA and protein expression levels of TGF-β1 following incubation for ≥6 h.

### High concentrations of mast cell chymase increase P-Smad2/3 and Smad2/3 protein expression, whereas low concentrations of mast cell chymase increase Smad7 protein expression

**Figure 3:**
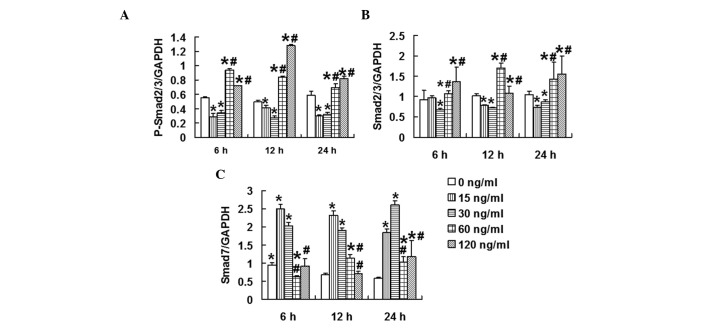
Protein expression of (A) P-Smad2/3, (B) Smad2/3 and (C) Smad7 in skin fibroblasts treated with chymase (0, 15, 30, 60 and 120 ng/ml) for 6, 12 and 24 h. Data are expressed as the mean ± standard deviation. ^*^P<0.05, vs. the 0 ng/ml concentration group; ^#^P<0.05, vs. the 15 and 30 ng/ml groups. P-Smad2/3, phosphorylated Smad2/3.

**Figure 4:**
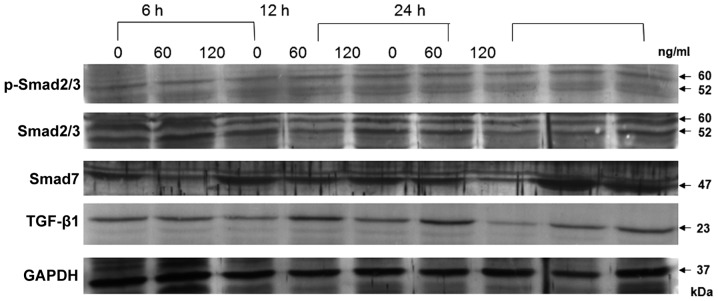
Western blots analysis showing the protein expression of P-Smad2/3, Smad2/3, Smad7, TGF-β1 and GAPDH in skin fibroblasts treated with chymase (0, 60 and 120 ng/ml) for 6, 12 and 24 h. TGF, transforming growth factor; GAPDH, glyceraldehyde-3-phosphate dehydrogenase; P-Smad2/3, phosphorylated Smad2/3.

To measure the protein expression levels of P-Smad2/3, Smad2/3 and Smad7, western blot analysis was performed. The assay revealed that the protein expression levels of P-Smad2/3 and Smad2/3 following incubation with chymase (60 and 120 ng/ml) for 6, 12 and 24 h were significantly higher compared with those in the control group (0 ng/ml; P<0.05). However, P-Smad2/3 and Smad2/3 protein expression levels following incubation with chymase at a concentration of 15 and 30 ng/ml were slightly lower compared with those in the control group (0 ng/ml; Figs. 3A, B and 4). By contrast, protein expression levels of Smad7 following incubation with 60 and 120 ng/ml chymase for 6 h were slightly lower compared with those in the control group (0 ng/ml), whereas Smad7 protein expression levels following incubation with 15 and 30 ng/ml chymase for 6 h were significantly higher compared with those in the control group (0 ng/ml) (P<0.05; Figs. 3C and 4). In addition, the protein expression levels of Smad7 following incubation with chymase (15, 30, 60 and 120 ng/ml) for 12 and 24 h were all higher compared with those in the control group (0 ng/ml). A statistically significant difference was observed in the Smad7 protein expression levels between those incubated with 15 and 30 ng/ml chymase and those incubated with chymase at 60 and 120 ng/ml (P<0.05; Figs. 3C and 4). These results indicated that high concentrations of mast cell chymase (60 and 120 ng/ml) increased P-Smad2/3 and Smad2/3 protein expression, whereas low concentrations of mast cell chymase (15 and 30 ng/ml) increased Smad7 protein expression.

## Discussion

One of the most important functions of mast cell chymase is the regulation of Ang II formation (14). Ang II directly acts on vascular smooth muscle to regulate the blood pressure and is associated with tissue fibrosis. In addition, chymase promotes the proliferation of fibroblasts in heart muscle and skin tissues (23–25), and promotes the release of TGF-β1 bound to the extracellular matrix (26). Furthermore, chymase is known to degrade procollagen for tissue remodeling (15,16) and participate in the inflammatory response (17,18).

A previous study demonstrated that chymase can activate the potential of TGF-β1. In cultured fibroblasts, chymase promotes the release of TGF-β1 from the bound protein (27). In human skin fibroblasts, chymase significantly increases the proliferation of fibroblasts; however, this process can be completely inhibited by chymase inhibitors, rather than Ang II receptor blockers (26). Chymase can facilitate the protein expression of TGF-β1 in fibroblasts; however, increased TGF-β1 levels can be inhibited by the chymase inhibitor, Suc-Val-Pro-Phep (OPh)_2_. Furthermore, an anti-TGF-β1 neutralizing antibody has been shown to completely inhibit chymase-induced cell proliferation, which may be mediated by the activation of TGF-β1 (28).

TGF-β is a secretory polypeptide signaling molecule that participates in a variety of pathophysiological processes in mammals. TGF-β affects cell proliferation and differentiation, and plays an important role in embryo development, extracellular matrix formation and immunoregulation. The subtype, TGF-β1, is closely associated with extracellular matrix deposition and fibrotic diseases, and is one of the main factors that affects wound healing and scar formation (29). In the present study, different concentrations of chymase were shown to affect the proliferation of skin fibroblasts in a dose- and time-dependent manner, which was consistent with the results of a previous study (19).

TGF-β1 mRNA expression levels in the skin fibroblasts following treatment with mast cell chymase (15 and 30 ng/ml) were higher compared with those in the control group; however, the protein expression levels following treatment with chymase at a concentration of 15 and 30 ng/ml were lower compared with those in the control group. In addition, TGF-β1 mRNA and protein expression levels following treatment with mast cell chymase at concentrations of 60 and 120 ng/ml were significantly higher compared with those following treatment with mast cell chymase at a concentration of 15 and 30 ng/ml.

Smads are signaling intermediates and antagonists of the TGF-β superfamily that are responsible for the intracellular signaling and regulation of TGF-β1 (7). The initial process of Smad pathway activation is R-Smad phosphorylation. Smad7 prevents the activation of R-Smad and downregulates TGF-β1 signaling. The TGF-β1/Smad signaling pathway plays an important role in skin development and wound healing (30–32). Chymase has been reported to induce myocardial fibrosis via the TGF-β1/Smad signaling pathway in cultured mouse cardiac fibroblasts (23). However, the effect of chymase on the TGF-β1/Smad signaling pathway in cultured human skin fibroblasts has not been reported previously.

In the present study, Smad2/3 and P-Smad2/3 protein expression levels in the fibroblasts treated with mast cell chymase (60 and 120 ng/ml) for 6, 12 and 24 h were higher compared with those in the control group. However, in the fibroblasts treated with 15 and 30 ng/ml chymase, the protein expression levels were lower compared with the control group. By contrast, Smad7 protein expression levels in the skin fibroblasts treated with 15 and 30 ng/ml chymase were higher compared with the control group and the 60 and 120 ng/ml chymase groups. The higher the TGF-β1 protein expression levels, the lower the Smad7 protein expression levels, and vice versa.

Phosphorylation of Smad2/3 is a key step in the activation of the Smad signaling pathway (33). The amount of P-Smad2/3 protein represents the degree of activation of the Smad signaling pathway. The aforementioned results demonstrated that chymase can activate the Smad signaling pathway. However, Smad proteins are not the only proteins activated by TGF-β (34,35), and may be associated with other signaling pathways. Thus, whether chymase is able to activate other signaling pathways requires further investigation.

Smad7 is the primary inhibitory protein in the TGF-β1 signaling pathway. The protein competitively binds to activated TGF-β receptor 1 to prevent the phosphorylation of R-Smad; thus, causing the downregulation of the Smad signaling pathway (36). In the present study, chymase at concentrations of 15 and 30 ng/ml significantly enhanced Smad7 protein expression, which was consistent with a previous study that reported TGF-β1 induced Smad7 protein expression in skin fibroblasts (37). Chymase can activate Smad2/3, and can increase Smad7 expression, forming an autocrine negative feedback loop. However, the expression of endogenous Smad7 induced by 60 and 120 ng/ml chymase was much lower compared with the protein expression levels of TGF-β1, Smad2/3 and P-Smad2/3, which promoted the Smad signaling pathway. The evidence that chymase (60 and 120 ng/ml) upregulated the mRNA and protein expression levels of TGF-β1 and promoted the phosphorylation of Smad2/3 protein indicated that chymase activated TGF-β1 and the intracellular signaling transduction Smad protein molecules, which promoted the transduction of the TGF-β1/Smad signaling pathway.

In conclusion, the present study investigated the effects of various concentrations of mast cell chymase on the TGF-β1/Smad signaling pathway in cultured skin fibroblasts. The results demonstrated that high concentrations of chymase facilitate the transduction of the TGF-β1/Smad signaling pathway.
